# Comparison of Health Level and Physical Activity between Korean and Other Countries Female College Students

**Published:** 2017-11

**Authors:** Tae-Young KIM

**Affiliations:** College of Education, Hankuk University of Foreign Studies, Seoul, Korea

## Dear Editor-in-Chief

Female College students in Korea are having changes in their bodies, transforming into more westernized shape from improvement of diet and improvements in their physique. Their average height and weight have gradually increased compared to 20 years ago. In 2016, the average height was 160.9 cm and the average weight was 57.1 kg for Korean female college students (1). As their Obesity Index is also on the rise, it is necessary to make comparison between Korean female college students and the foreign female college students (Asia: 97 Students / Europe: 46 Students / South America: 20 Students).

For this research, total of 389 female college students, 226 from Korea and 163 from overseas participated through survey. Their average age was 21.5 yr old, average height 162.0 cm and average weight of 54.6 kg. Compared to their adolescence, college students are more easily exposed to harmful behaviors such as unhealthy diet, sedentary lifestyle, smoking cigarette and binge drinking (2–3), and they often experience mental illness such as schizophrenia or bipolar disorder (4). Hence, it is essential to maintain a good health during college years where a fundamental strength is formed to become socially active. The study was approved by Ethics Committee of the university.

In addition, many college students do not consider it matters even though their college years to be an important period to build up a long-term foundation of health (5). They no longer continue self-restrained lifestyle as they exit from adolescence which leads to obesity (6); therefore, consistent physical activities must be strongly encouraged to them during this period to prevent being obese.

In recent years, Korean female college students are having more interest in seeking for easier methods to decrease body fat and outer beauty rather than being involved in physical activities. It is speculated that there will be differences between Korean and other countries female college students in the same age group. Table 1 shows the study result of surveying the health level and physical activity level between Korean and other countries female college students. Statistical significance of this study was set at *P*<0.05 using SPSS Window version 18.0 (Chicago, IL, USA). Other countries female college students recorded higher significance level (*P*<.001) than Korean female college students in health level, indicating that other countries female college students have higher interest in health related standards. Other countries female college students also had higher significance level (*P*<.001) in exercise preferences and exercise status than Korean students, meaning that other countries students possess higher willingness to exercise.

**Table 1:** Health level and Physical activity levels of Korea and other countries female college students

***Item***	***Group***	***Number***	***Mean***	***SD***	***t***	***df***	***P***
Health level	KFC	226	3.51	0.84	−5.905	373.13	.001
	OCFC	163	3.98	0.73			
Exercise preferences	KFC	226	3.26	0.97	−3.450	387	.001
	OCFC	163	3.59	0.85			
Exercise Status	KFC	226	2.07	0.83	3.440	387	.001
	OCFC	163	1.77	0.83			
Physical activity (times/week)	KFC	226	1.26	0.48	−3.457	315.98	.001
	OCFC	163	1.45	0.56			
Exercise duration (months)	KFC	70	13.27	17.46	−4.160	100.35	.001
	OCFC	79	37.43	48.17			

KFC: Korean female college, OCFC: Other country female college, ^***^P<.001

The result of studying whether sufficient physical activities achieved shows that other countries female college students are more actively involved in physical activities by recording higher significant level (*P*<.001) than Korean female college students. Furthermore, among total of 149 students (70 Korean, 79 Foreign) who exercised more than once a month, the comparison on these two groups showed that other countries female college students had three times significantly longer exercise duration level (*P*<.000) than Korean female students, as the other countries female college students persisted for 3 years and 1 month while Korean female college students lasted 1 year and 1 month. However, no significant differences were seen in exercise frequency, single exercise time and sedentary time between the two groups.

In conclusion, other countries female college students are more active and willing to maintain good health from exercise compared to Korean female college students. Current Korean female college students value outer beauty rather than physical health. Therefore, it is important to change Korean female college students’ perspective on beauty. It is necessary to provide Korean female college students with effective programs to improve their health level and more information guide within campuses for better accessibility in the future. Moreover, it would be desirable to keep students in the habit of sharing and planning health improvement plans and actively participating in physical exercises.

## References

[1] Ministry of Education (2017). 2016 Students Health Examination Sample Statistics Analysis.

[2] Lau JS Adams SH Irwin CE Jr Ozer EM (2013). Receipt of preventive health services in young adults. J Adolesc Health, 52:42–9. 23260833 10.1016/j.jadohealth.2012.04.017 PMC3574866

[3] Ozer EM Urquhart JT Brindis CD (2012). Young adult preventive health care guidelines: there but can’t be found. Arch Pediatr Adolesc Med, 166:240–7. 22393182 10.1001/archpediatrics.2011.794

[4] Freudenberg N Manzo L Mongiello L (2013). Promoting the health of young adults in urban public universities: a case study from City University of New York. J Am Coll Health, 61:422–30. 24010497 10.1080/07448481.2013.823972

[5] Joh HK Kim HJ Kim YO Lee JY Cho BL Lim CS Jung SE (2017). Health promotion in young adults at a university in Korea: A cross-sectional study of 625 participants in a university. Medicine (Baltimore), 96(7):e6157. 28207551 10.1097/MD.0000000000006157 PMC5319540

[6] Lee H Lee D Guo G Harris KM (2011). Trends in body mass index in adolescence and young adulthood in the United States: 1959–2002. J Adolesc Health, 49: 601–8. 22098770 10.1016/j.jadohealth.2011.04.019 PMC3228354

